# 

**Published:** 1888-10-13

**Authors:** 


**To Residents, Secretaries, and Matrons.**

The Editor will be glad to have the Regulations and each Report as issued by Asylums, Hospitals, Convalescent and Nursing Institutions, as well as those of other Charities, and an early copy in duplicate of all Appeals and Festival Notices, etc., addressed to *The Editor, The Lodge, Porchester Square, London, W.* Any superintendent, secretary, matron, or other chief official who regularly sends such information may be registered, on application to the Publisher, to receive free of cost a cover for binding The Hospital in half-yearly volumes.

**Notice to Advertisers and Subscribers.**

All Advertisements and Business Communications should be addressed to The Publisher, *not to the Editor*, The Hospital, 38, Old Jewry, E.C.
A Scale of Charges will be found in the Advertisement Sheets, page iii.

### Amusmements and Relaxation.

### NEW PUZZLE PRIZE SCHEME.

COMMENCED OCTOBER 6th, ENDS DECEMBER 29th, 1888.

On October the 6th a New Prize Scheme was commenced in these columns.

Prizes will be awarded for the best Original Puzzles in Prose or Verse relating to any of the Social, Philanthropic, or Political topics of the day, and taking the form of Double Acrostics, Anagrams, Square Words, Decapitations, Transpositions, or any other Puzzles which the ingenuity of the competitors may suggest. (*Answers must accompany contributions.*)

*Readers* are requested to send in weekly the Number of Marks they consider each composition to be entitled to, three being the highest score for any one contribution.

Eight Prizes of Standard Books will be given, the choice of the book to be left to the winner.

First, Second, Third, Fourth, and Fifth Prizes for best Original Puzzles, of £1 1s., 15s., 10s. 6d., 7s. 6d., and 5s. each.

First, Second, and Third Prize for Solutions of 15s., 10s. 6d., and 5s. each.

N.B.—All contributions must reach the office not later than the *First Post* on *Thursday* in each week.

*Puzzles for solution will be given next week, when we hope to have received many contributions from both* old *and* new *competitors.*

### THE CHILDREN'S CORNER.

During the past year we have offered the following

PRIZES

FOR MOST CORRECT ANSWERS TO PUZZLES.

One Pound a Month.

A PRIZE OF THREE GUINEAS

to the Competitor who shall have sent in the largest number of correct or best answers during the twelve months.

*In future these competitions will be discontinued.*

### Results of Fifth Week Puzzles.

No. 17. Double Acrostic —

| | | |
|---|---|---|
| N | e | W |
| A | re | A |
| P | i | T |
| O | n | E |
| L | oite | R |
| E | e | L |
| O | rinoc | O |
| N | egr | O |

No. 18. Enigma.—Chair.

No. 19. Flowers Enigmatically Expressed.—1. Ragged-Robin. 2. London Pride. 3. Sunflower. 4. Bitter Sweet.

No. 20. Geographical Charade.—Manchester.

| Marks. | Sept. 29th. | Total First, Second, Third, Fourth, and Fifth Weeks. |
|---|---|---|
| A. C. K. | 4 | 19 |
| Gem | 3 | 17 |
| G. M. M. | — | 8 |
| Scrooge | 4 | 15 |
| Thanet | 4 | 19 |
| Tynesider | — | 3 |
| Judy | 4 | 13 |
| Primrose | 4 | 19 |
| J. W. M. | 4 | 16 |
| Agamemnon | 4 | 19 |
| G. E. R. | 4 | 18 |
| Happy Eliza | 4 | 13 |
| Penelope | 4 | 15 |
| Boadicea | 4 | 18 |
| Ikey Mo | — | 5 |
| Poodle | — | 2 |

We are glad to find that the competition for the last month has been even a closer one than usual, A. C. K., Thanet, Primrose, and Agamemnon each scoring 19 marks. We therefore divide the Prize of One Pound equally between

"Thanet," Miss WINNIE CHEXFIELD,
R. S. B. Infirmary, Margate.

"Primrose," Master STAFFORD ASTON,

8, Elsworthy Terrace, Primrose Hill, N.W.

"Agamemnon," Master H. H. HULL,
The Limes, Boxmoor, Herts.

### Results of Yearly Competition.

| Names. | Total Marks. | Names. | Total Marks. |
|---|---|---|---|
| 1. A. C. K. | 200 | 44. Bunny | 16 |
| 2. Scrooge | 176 | 45. Military Cadet | 16 |
| 3. Gem | 146 | 46. P. S. | 15 |
| 4. Judy | 105 | 47. Alert | 15 |
| 5 Happy Eliza | 98 | 48. Oswald H. | 15 |
| 6. Plummy Fluff | 89 | 49. Moina | 15 |
| 7. Spider | 87 | 50. Bookworm | 14 |
| 8. A. G S. McCulloch | 86 | 51. Toad | 14 |
| 9. Mount Edgcumbe | 84 | 52. The Eda | 14 |
| 10. Alsie | 78 | 53. Dame Durden | 13 |
| 11. Jumps | 61 | 54. Good Old Killaloe | 13 |
| 12. Excelsior | 61 | 55. Jack | 13 |
| 13. Thanet | 61 | 56. The Young Canadian | 12 |
| 14. Tim | 55 | 57. Pickles | 12 |
| 15. Poodle | 54 | 58. Gerald H. | 11 |
| 16. Pepita | 53 | 59. Young Surgeon | 11 |
| 17. Hercules | 44 | 6c. Jumbo | 10 |
| 18. Squeaker | 40 | 61. Holmes | 10 |
| 19 Boadicea | 38 | 62. Princess Ida | 10 |
| 20 Skye | 36 | 63. Punch | 9 |
| 21. Ossian | 36 | 64. Jacko | 9 |
| 22. M. S. W. | 36 | 65. Babette | 8 |
| 23. R. E. Herbertson | 31 | 66 W. R. | 7 |
| 24. Jerusalem | 29 | 67. Janette | 7 |
| 25. Nunkey | 28 | 68. Umslopogaas | 6 |
| 26. Paddy | 28 | 69. A. T. | 6 |
| 27. Fern | 27 | 70. H. E. W. | 6 |
| 28. Toby | 27 | 71. Faith | 6 |
| 29. Geranium | 25 | 72. Ikey Mo | 5 |
| 30. Robinson | 25 | 73. Snap | 5 |
| 31. G. M. M. | 23 | 74. Uncus | 4 |
| 32. Druce | 22 | 75. Paul Methven | 4 |
| 33. G. B. P. | 22 | 76. Alma | 4 |
| 34. Primrose | 19 | 77. Tyke | 4 |
| 35. Agamemnon | 19 | 78. M. M. S. | 4 |
| 36. G. E. R. | 18 | 79. Kelpie | 4 |
| 37. Curley | 18 | 80. Inanity | 4 |
| 38. Penelope | 18 | 81 Mia Caro | 4 |
| 39. Betty Burke | 18 | 82. Genaro | 4 |
| 40. Bagshot | 17 | 83. Sambo | 3 |
| 41. Kensington | 17 | 84. Baby | 3 |
| 42. Tynesider | 17 | 85. Ellie | 3 |
| 43 J. W. M. | 16 | 86 Joe Pena | 2 |

Above we have given the names and full score of marks gained by each competitor who has entered for the Children's Corner competitions since October, 1887, and it will be seen that A. C. K. has succeeded in winning the Three Guinea Prize for largest number of correct solutions to puzzles during the past twelve months. We therefore forward the Prize to

"A. C. K," Master ARTHUR CLIFTON KELLY,
Ellesmere, Gratwicke Road, Worthing.

### Conditions.

I. Every paper sent in must be clearly written, and one side only must be used. All competitors must mark at the head of their papers the number of their competition.

II. Each paper must be signed by the author with his or her real name and address. A *nom de plume* may be added if the writer does not desire to be referred to by us by his real name. In the case of all prizewinners, however, the real name and address will be published.

III. All communications relating to prize competitions should be addressed to "The Prize Editor, The Hospital, 38, Old Jewry, London, E.C." Competitors are requested not to include in letters, etc., to the Prize Editor communications on matters of other business. All letters must be fully prepaid.

IV. The Prize Editor of The Hospital is the sole judge of the competitions, and his decision is in all cases final and without appeal.

V. The Prize Editor reserves the right to publish any paper sent in, whether it receives a prize or not. He does not hold himself responsible for any MSS. sent, nor can he undertake to return rejected competitions.

